# Interstitial *de novo* 18q22.3q23 deletion: clinical, neuroradiological and molecular characterization of a new case and review of the literature

**DOI:** 10.1186/s13039-016-0285-1

**Published:** 2016-10-10

**Authors:** Elisa Tassano, Mariasavina Severino, Silvia Rosina, Riccardo Papa, Domenico Tortora, Giorgio Gimelli, Cristina Cuoco, Paolo Picco

**Affiliations:** 1 Laboratorio di Citogenetica, Istituto Giannina Gaslini, L.go G.Gaslini 5, 16147 Genoa, Italy; 2 Neuroradiology Unit, Istituto Giannina Gaslini, Genoa, Italy; 3 Istituto Giannina Gaslini, Genoa, Italy

**Keywords:** 18q- syndrome, Array CGH, Brain MRI, Spectroscopy, Diffusion tensor imaging, Radial diffusivity

## Abstract

**Background:**

Deletions of the long arm of chromosome 18 cause a common autosomal syndrome clinically characterized by a protean clinical phenotype.

**Case presentation:**

We report on a 16-month-old male infant affected by fever attacks apparently unrelated with any infectious or inflammatory symptoms, growth retardation, bilateral vertical talus, congenital aural atresia, dysmorphisms, mild psychomotor delay, and peculiar neuroradiological features. Array-CGH analysis revealed one of the smallest 18q22.3q23 interstitial deletions involving five genes: *TSHZ1*, *ZNF516*, *ZNF236, MBP,* and *GALR1*.

**Conclusions:**

Herein we focus on previously unreported heralding symptoms and neuroradiological abnormalities which enlarge the spectrum of 18q deletion syndrome demonstrating that a small deletion can determine a complex phenotype.

## Background

Deletions of the long arm of chromosome 18 (18q-) described by De Grouchy et al. [1] cause a common autosomal syndrome (1:40,000 live births) [2] characterized by a wide range of phenotypic abnormalities related to the size of the deletion and the position of breakpoints. Common clinical features of the 18q- syndrome are short stature, facial dysmorphisms, foot deformities, congenital aural atresia without microtia, variable intellectual disability, cerebral white matter abnormalities, and microcephaly [3, 4]. Less commonly reported features are kidney malformations, bone dysplasia, growth hormone deficiency, congenital heart disease (atrial or septal defect), and skin manifestations (dimpling and excessive whorls on fingertips) [5]. Besides cognitive impairment, neurological abnormalities may include hypotonia, seizures, nystagmus, poor coordination, tremor, and choreoathetosis.

Several studies tried to define the specific regions involved in each phenotypic trait and to identify possible candidate genes [4, 6–11].

The advent of high-resolution microarray comparative hybridization (aCGH) has allowed more precise phenotype/genotype correlations of such protean syndrome. Interestingly, aCGH analysis performed in a series of 189 patients with deletions of chromosome 18q revealed that none of the unrelated individuals shared the same breakpoints, demonstrating a high level of variability and genomic complexity surrounding deletions of 18q [12].

Herein we report a patient with one of the smallest 18q22.3q23 interstitial deletions described so far, focusing on the heralding symptoms and the advanced neuroradiological findings. These factors, together with genetic assessment, can improve our knowledge about this group of patients.

## Case presentation

**Fig. 1:**
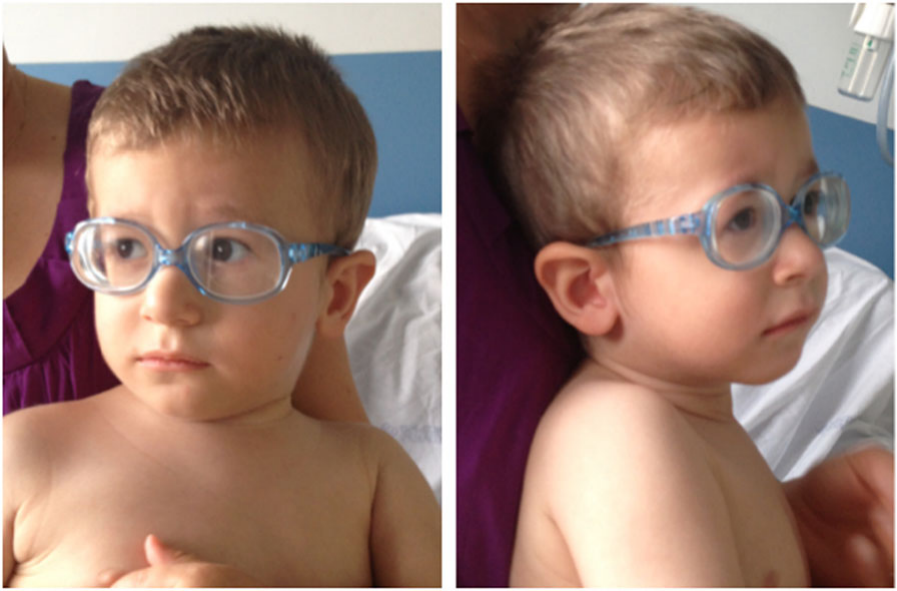
Picture of the patient performed at 14 months demonstrates epicanthal folds, smooth philtrum, thin upper lip, low-set ears, prominent frontal bossing and sparse light hair, carp-shaped mouth

We report the case of a 2-year-old male born at term after an uneventful pregnancy from healthy unrelated parents (Fig. 1). The family history was unremarkable. At birth his weight was 2.990 kg (10th centile), length was 48 cm (6th centile), and head circumference was 33 cm (6th centile). Apgar scores were 8 and 9. Bilateral congenital vertical talus was present at birth. Motor development was delayed (autonomous sitting acquired at 13–14 months of age). From the age of 5 months, the patient presented long-term fever attacks with a continuous/sub-continuous pattern and spikes up to 40–41 °C, apparently not associated with any infectious or inflammatory manifestation. Acute phase reactants were persistently within normal ranges. The only reported concomitant symptoms were hyporexia and poor weight gain. Neither antipyretic nor antibiotic treatment seemed to be effective.

**Fig. 2:**
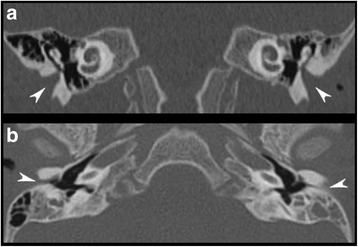
Temporal bone CT. Coronal (**a**) and axial (**b**) images reveal marked stenosis and peculiar vertical orientation of the bony portions of the external auditory canals (arrowheads) associated with rotation and fusion of the malleus to the lateral middle ear cavity wall

At the age of 16 months the patient was admitted to our Unit for fever of unrecognized origin. Physical examination revealed growth impairment: weight was 7.83 kg (<3th centile), length was 73 cm (<3th centile, SDS −2.5) with a BMI of 14.7 (−2.3 SDS). Mild dysmorphic features were present (i.e. frontal bossing, low-set ears), dental eruption was regular although he showed mild organ enamel dysplasia. Neurologic assessment showed global hypotonia, hyperlaxity, hyporeflexia, and motor delay (he could only walk with support). During hospitalization, we documented recurrent abrupt fever spikes (up to 39 °C) lasting 3–5 days, without any other systemic or local sign of inflammation or infection. Fever attacks were well tolerated; skin hyperthermia and sweating were present too. Laboratory investigations showed inflammatory parameters within normal ranges also during fever spikes, mild microcytic anaemia (Hb 11.8 g/dl, MCV 72 fL), partial IgA deficiency (27 mg/dl) with normal IgM and IgG, normal lymphocyte subpopulations, and normal immune response against vaccines. The infectious disease workout was negative: only once a low degree bacteriuria (below 50.000 UFC/ml) was incidentally found and antibiotic therapy (amoxicillin-clavulanic acid) was started without any efficacy against the fever. Abdominal and renal tract ultrasound scan, cystomanometry, renal scintigraphy and voiding cystography, chest X-ray, echocardiography, and single-contrast esophagogastroduodenal X-ray were also normal. Thyroid hormones, acylcarnitine profile, plasma lactate, urinary catecholamine, organic acid, and mevalonate urinary concentrations resulted within normal ranges. Brain MRI showed diffuse white matter signal hyperintensity on T2-weighted images in keeping with delayed myelination. Visual evoked potentials showed latency of cortical components at the upper limit of normal ranges, and somatosensory evoked potentials revealed a conduction velocity at the upper limit of normal ranges for age with regular latency of cortical, subcortical, cervical, and peripheral components in the upper limbs. Ophthalmologic evaluation revealed high degree myopia. Otologic examination disclosed stenosis of the external auditory canals without structural abnormalities of the pinna. Behavioural hearing assessment demonstrated bilateral hearing impairment. CT scan of the temporal bone confirmed bilateral atresia of the membranous portions and marked stenosis of the bony portions of the external auditory canals (Fig. 2).

**Fig. 3:**
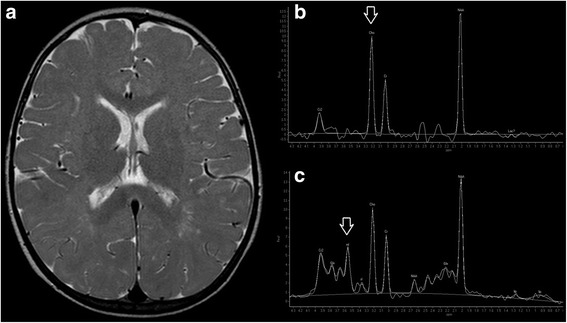
Brain MRI and MR spectroscopy performed at 24 months of age. **a** Axial T2-weighted image shows diffuse hyperintensity of the cerebral white matter with relative sparing of the corpus callosum. Long TE spectroscopy (**b**) reveals an elevated choline peak (arrow), while short TE spectroscopy (**c**) demonstrates a slightly increased myoinositol peak (arrow)

At the age of 24 months, the patient was re-evaluated. Growth impairment persisted: weight was 8.5 kg (<3th centile), length was 82 cm (SDS −1.5) with a BMI of 12.6 (−4.3 SDS); psychomotor delay persisted (e.g. walking was possible with an enlarged basis, no verbal expression was reported). Notably, fever attacks had spontaneously disappeared from the age of 20 months. A follow-up brain MRI at this age demonstrated an unchanged pattern of abnormal intensity of the white matter on T2-weighted images (Fig. 3a).

## Results

### Genetic study

**Fig. 4:**
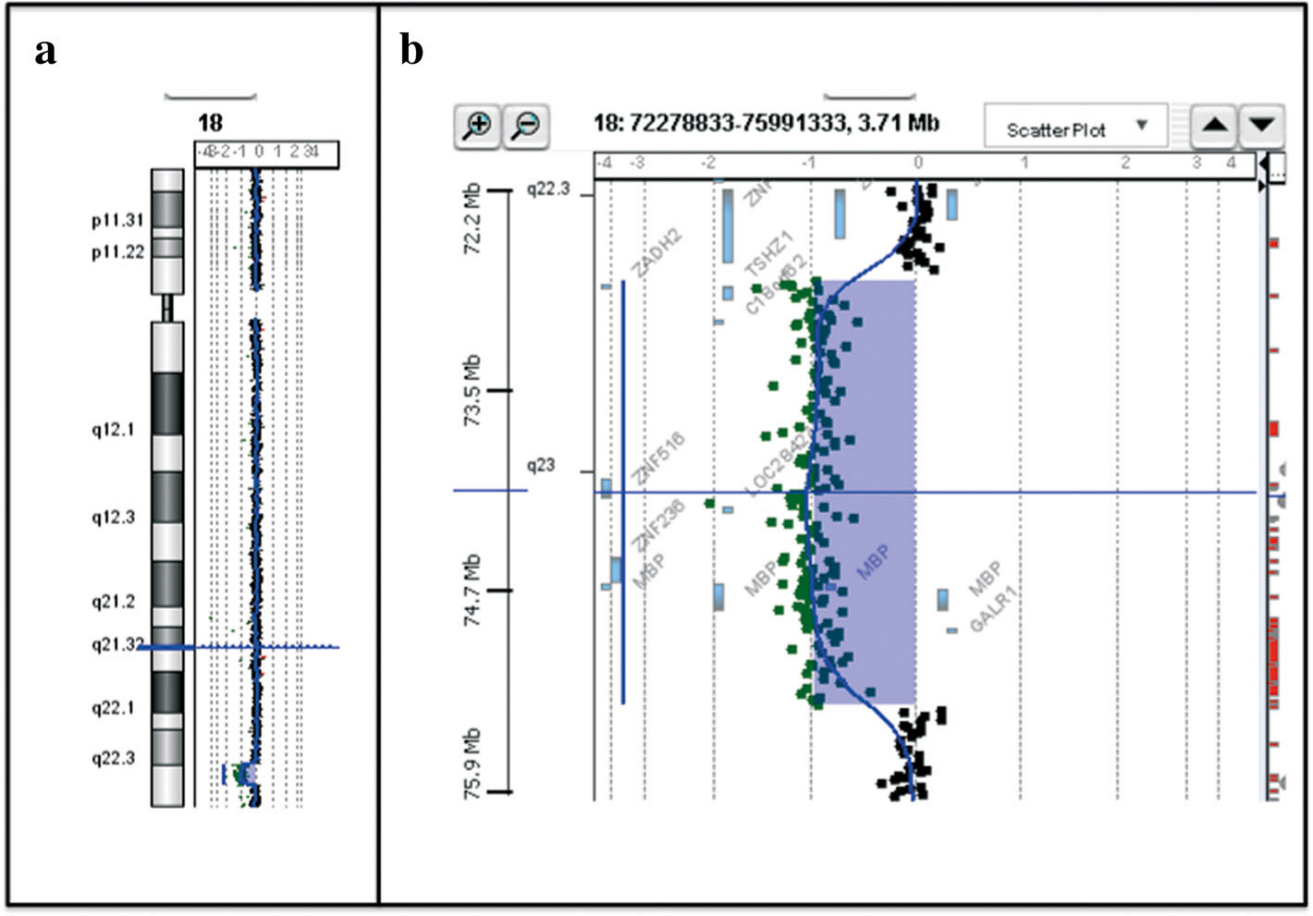
Results of array-CGH analysis. **a** Chromosomal view. **b** Zoom view of long arm of chromosome 18 shows a 2.5 Mb deletion at 18q22.3q23 spanning from position 72,887,342 bp (clone A_16_P20921235) to 75,418,267 (clone A_16_P41107360) flanked by probe A_16_P20921038 (72,818,369 bp) and probe A_14_P133188 (75,452,546 bp) according to UCSC Genome Browser (hg19; GRChBuild 37.1, February 2009)

Array-CGH analysis of the patient showed an interstitial deletion at 18q22.3q23 spanning about 2.5 Mb of genomic DNA from position 72,887,342 bp (clone A_16_P20921235) to 75,418,267 (clone A_16_P41107360) flanked by probe A_16_P20921038 (72,818,369 bp) and probe A_14_P133188 (75,452,546 bp) according to UCSC Genome Browser (GRCh37/hg19 assembly) (Fig. 4). The deleted region contains 5 OMIM genes: *TSHZ1* (MIM 1614427, Teashirt zinc finger homeobox), *ZNF516* (MIM 615114, Zinc finger protein 516), *ZNF236* (MIM 604760, Zinc finger protein-236), *MBP* (MIM 159430, Myelin basic protein), *GALR1* (MIM 600377, Galanin receptor 1). Array-CGH of the parents was normal.

### Neuroimaging data

Analysis of the DTI scalars revealed slightly lower fractional anisotropy and higher mean, radial, and axial diffusivity in the patient compared to controls in all the assessed white matter regions. However, these differences were not statistically significant as assessed by non-parametric Mann–Whitney test (Table 1).

**Table 1:** Diffusion Tensor Imaging data in the present patient compared with 5 age-matched controls

	Mean fractional anisotropy (SD)		Mean diffusivity (x10^−3^) (SD)		Mean radial diffusivity (x10^−3^) (SD)		Mean axial diffusivity (x10^−3^) (SD)	
	Patient	Controls	Patient	Controls	Patient	Controls	Patient	Controls
CS	0.300 (0.091)	0.349 (0.019)	1.000 (0.100)	0.920 (0.044)	0.800 (0.100)	0.720 (0.044)	1.300 (0.100)	1.240 (0.054)
CC	0.622 (0.149)	0.637 (0.051)	1.100 (0.100)	0.920 (0.044)	0.700 (0.200)	0.520 (0.044)	1.900 (0.300)	1.700 (0.052)
PLIC	0.555 (0.115)	0.639 (0.017)	0.900 (0.100)	0.840 (0.054)	0.600 (0.100)	0.480 (0.044)	1.600 (0.200)	1.520 (0.109)
CWM	0.655 (0.069)	0.596 (0.087)	0.800 (0.100)	0.780 (0.054)	0.500 (0.100)	0.460 (0.083)	1.500 (0.200)	1.400 (0.173)
Pons	0.364 (0.135)	0.427 (0.063)	0.900 (0.100)	0.800 (0.030)	0.700 (0.100)	0.580 (0.044)	1.200 (0.100)	1.200 (0.070)

Legend: *CC* corpus callosum, *CS* centrum semiovale, *CWM* cerebellar white matter, *PLIC* posterior limb of internal capsule, *SD* standard deviation

MRS demonstrated normal N-acetyl aspartate, absent lipids and lactate, and increased choline peak on the long TE sequence with N-acetyl aspartate/creatine and choline/creatine ratios of 2.18 and 1.79, respectively. On the short TE sequence, slight increase of the myoinositol peak was noted with N-acetyl aspartate/creatine and choline/creatine ratios of 2.12 and 1.44, respectively (Fig. 3b, c).

## Discussion

Here we describe the peculiar clinical phenotype and neuroradiological features of a child harbouring an uncommon 18q22.3q23 interstitial microdeletion.

To our knowledge, this is one of the smallest 18q interstitial deletions described so far, including five OMIM genes: *TSHZ1*, *MBP*, *ZNF516*, *GALR1,* and *ZNF236*. Among these genes, *TSHZ1* and *MBP* were considered dosage-sensitive either with high or low penetrance of the abnormal phenotype, the haploinsufficiency of *ZNF516* and *GALR1* seemed unrelated with the clinical manifestations of 18q- syndrome, while the consequences of a copy number change of *ZNF236* are still unknown [5] (http://www.pediatrics.uthscsa.edu/centers/Chromosome18/dosage.asp). Only three other patients harbouring a very similar deletion and similar breakpoints have been reported [[3, 12], case 1; [13], case 2]. Phenotypically, all showed dysmorphic features and CAA, three patients showed intellectual disability and only two a growth retardation (Table 2). Interestingly, all of them shared the deletion of *TSHZ1* and *MBP* genes.

**Table 2:** The peculiar clinical phenotype and neuroradiological features of a child harbouring an uncommon 18q22.3q23 interstitial microdeletion

Patients (coordinates)	Growth retardation	Dysmorphisms	CAA	Dysmielination	Intellectual disability	CVT	Others	Genes
Present case Arr cgh18q22.3q23 (chr18:72887342–75418267) 2.531 Mb	Yes	Epicanthal folds, long nasal philtrum, thin upper lip, low-set ears, prominent frontal bossing and sparse light hair	Yes	Yes	Yes	Yes	Recurrent fever	ZADH2 TSHZ1 SMIM21 ZNF516 ZNF236 MBP GALR1
[12] Arr cgh 18q22.3q23 (chr18:70508757–74627485) 4.118 Mb	Yes	bitemporal narrowing, abnormal external ears	Yes	Not reported	Yes	No	Cleft soft palate	ZNF407 ZADH2 TSHZ1 SMIM21 ZNF516 ZNF236 MBP GALR1
[3] pat.1 Arr cgh 18q22.3q23 (chr18:72493281–74110482) 1.617 Mb	No	prominent epicanthal folds, depressed nasal bridge,	Yes	No	Yes (Speech delay)	Yes		ZNF407 ZADH2 TSHZ1 SMIM21 ZNF516
[13] pat.2 Arr cgh 18q22.3q23 (chr18:72.9–75.4) 2.5 Mb	No	hypertelorism, midfacial hypoplasia, and a broad mouth with prominent lips	Yes	Not reported	No	Yes	Two sons with the same clinical features of the mother	TSHZ1 SMIM21 ZNF516 ZNF236 MBP GALR1

*TSHZ1* gene is a member of the teashirt-type zinc-finger protein family encoding putative zinc finger transcription factors. Targeted inactivation in mouse resulted in a neonatal lethal phenotype with soft palate clefting, vertebral malformations, and abnormalities of the middle ear [14]. In particular, the inactivation of *Tshz1* seemed to lead to the deregulation of *Cbfa1* (*RUNX2*) in mesenchymal condensation, which allows the development of the tympanic ring. Therefore, the hemizygosity of *TSHZ1* gene was indicated as a possible candidate for aural atresia in humans [13]. Recently, it has been demonstrated that deletion of *TSHZ1* in mice leads to olfactory bulb hypoplasia and severe olfactory impairment [15]. This gene has been proposed as a candidate gene for congenital vertical talus (CVT) in a study on three patients with 18q deletions [3]. Notably, congenital aural atresia without microtia and CVT were also found in our patient, thus supporting the hypothesis of a pivotal role of *TSHZ1* in the development of ears and feet [[3, 12], case 1, [13], case 2].

*MBP* gene encodes a myelin basic protein that represents one of the most important structural proteins of the myelin sheath. In particular, *MBP* is thought to play a major role in myelin compaction. Brain MRI studies in patients with 18q- syndrome typically showed diffuse white matter T2 hyperintensity with poor differentiation between gray and white matter [16, 17]. These findings have been interpreted as delayed or reduced myelin formation related to haploinsufficiency of the *MBP* gene. However, the precise pathogenetic mechanisms underlying white matter abnormalities in 18q- syndrome remain poorly understood. Loss of both *MBP* genes in homozygous *shiverer (shi)* mice was histologically characterised by almost complete absence of myelin in the central nervous system [18]. However, heterozygous *shiverer* mice were found clinically normal with normal myelin on brain MRI and pathological examination [19, 20]. Interestingly, a recent autopsy study on a 6-year-old boy with 18q- syndrome and abnormal white matter on brain MRI revealed prominent astrogliosis with normal myelin fibers and compact myelin sheaths [21]. Immunohistological examination revealed normal MBP immunoreactivity in myelinated fibers, but reduced immunoreactivity in oligodendrocytes. The authors therefore suggested that the signal abnormalities exhibited in 18q-syndrome could reflect astrogliosis rather than hypomyelination [21]. This hypothesis is supported by the increase in choline, creatine, and myoinositol concentrations on MR spectroscopy found in two patients with 18q- syndrome [22, 23], as well as in the present patient. These findings suggest functional abnormalities of oligodendrocytes resulting in accelerated myelin turnover associated with astrogliosis. Indeed, MR spectroscopy in patients with Pelizaeus-Merzbacher disease, a representative hypomyelination disorder, typically shows decreased choline due to absence of myelin and mature oligodendrocytes [24].

Diffusion tensor imaging (DTI), an advanced MRI technique assessing the diffusion properties of white matter water in vivo, has been used in the evaluation of several congenital and acquired white matter disorders [25]. On DTI, normal white matter tracts show high diffusion anisotropy (fractional anisotropy, FA) with higher diffusivity in the direction parallel to the fibers (axial diffusivity, AD) than in the direction perpendicular to them (radial diffusivity, RD). Increased RD values were detected in mouse models of hypomyelination [20, 26, 27] and were interpreted as an indicator of myelin density, since the absence of myelin facilitates movement of water molecules perpendicular to the axons. Interestingly, also human DTI studies indicated RD to be the most sensitive among DTI parameters in distinguishing patients with hypomyelinating disorders from controls [25, 28]. To the best of our knowledge, DTI data have never been reported in patients with 18q- syndrome. Intriguingly, in the present patient, we did not find a significant increase in RD compared to controls, which reinforces the hypothesis of absent disruption of myelin formation and/or compaction in this disorder. In this regard, the consequences of haploinsufficiency of the *MBP* gene in humans remain unknown and further investigations are needed to elucidate the role of the astroglia in 18q- syndrome.

*GALR1* gene is involved in regulation of appetite and pituitary hormone secretion, memory, learning, and anxiety [29]. Intriguingly, this gene encodes the galanin receptor GALR1 that mediates the hyperpolarisation of the warm sensitive neurons in the preoptic area of the hypothalamus. Warm sensitive neurons are involved in changes of core body temperature during the fever response [30]. Hence, we could speculate whether the recurrent fevers in the present patient might be secondary to GALR1 deficiency or dysfunction. Although our patient showed teeth abnormalities suggestive of hypohidrotic ectodermal dysplasia, no other clinical signs of this disease were present. Notably ectodermal dysplasia-like findings have been reported only in another child with 18q- syndrome [31]. So far, we are unable to state if the thermic dysregulation in our patient is an occasional finding or a 18q- syndrome-related symptom: obviously further studies on larger case series need to confirm this association.

*ZNF516* and *ZNF236* are zinc finger transcription factors that could have important roles in the regulation of other genes, but their influence in determining the phenotype of our patient is still unclear.

## Conclusion

In conclusion, the clinical features of our patient enlarge the phenotypical spectrum of 18q deletion syndrome, suggesting that even such small deletion can determine a complex phenotype. Furthermore, the use of advanced brain MRI technique, including DTI and MR spectroscopy, may help to define the type of white matter involvement and therefore improve our knowledge about this interesting syndrome.

## Materials and methods

### Array-CGH analysis

Array-CGH analysis was performed on the patient and his parents using Human Genome CGH Microarray Kit G3 180 (Agilent Technologies, Palo Alto, USA) with ~13 Kb overall median probe spacing. Labelling and hybridization were performed following the protocols provided by the manufacturers. A graphical overview was obtained using Agilent Genomic Workbench Lite Edition Software 6.5.0.18.

### Neuroimaging data

Diffusion Tensor Imaging (DTI) and MR spectroscopy (MRS) studies were performed during the last MRI follow-up on a 1.5 Tesla scanner (Achieva, Philips Medical Systems, Best, The Netherlands). DTI data were acquired using a single-shot spin-echo echoplanar imaging diffusion sequence in 32 directions with TR/TE: 9318 ms/71 ms, FOV: 224x224x120 mm, flip angle: 90°, 60 slices, acquisition voxel size: 2 × 2 × 2 mm, slice thickness: 2 mm, slice gap: 0 mm, 2 b values of 0 and 800. DTI data were processed using the FSL software package (Analysis Group FMRIB, Oxford, United Kingdom) [32]. The fractional anisotropy (FA), mean diffusivity (MD), radial diffusivity (RD), and axial diffusivity (AD) values were calculated with a ROI-based approach in bilateral centrum semiovale, posterior limbs of internal capsule, corpus callosum, cerebellar white matter, and pons using MIPAV (https://mipav.cit.nih.gov/). These values were compared with those obtained from 5 healthy age-matched infants with normal MRI who were studied for headache, first seizure, or minor trauma.

MRS with long and short TE was performed with the point resolved excitation spin-echo sequence (PRESS sequence; long TE: TR/TE = 2000/144 ms; short TE: TR/TE = 2000/35 ms; voxel size 8 ml; 512 samples, 128 NSA). The measuring voxel was placed in the parieto-occipital white matter.

## Abbreviations

(18q-): Deletions of the long arm of chromosome 18
aCGH: High-resolution microarray comparative hybridization
DTI: Diffusion tensor imaging
MRS: Magnetic resonance spectroscopy
